# Positive fluid balance and prognostic factors of ICU mortality in patients admitted with septic shock

**DOI:** 10.1186/cc14073

**Published:** 2014-12-03

**Authors:** W Koonrangsesomboon, B Khwannimit

**Affiliations:** 1 Department of Internal Medicine, Faculty of Medicine, Prince of Songkla University, Hat Yai, Songkhla, Thailand; 2 Division of Critical Care Medicine, Department of Internal Medicine, Faculty of Medicine, Prince of Songkla University, Hat Yai, Songkhla, Thailand

## Introduction

The amount of fluid during resuscitation of septic shock is important. Too little fluid may result in tissue hypoperfusion; however, too much fluid may result in volume accumulation. Several recent studies have demonstrated that a positive fluid balance in critical illness is associated with deteriorating outcomes. However, some studies have shown opposite results. The objective of this study was to determine whether initial fluid balance in septic shock patients is correlated with ICU mortality.

## Methods

This is a retrospective study of septic shock patients admitted to a mixed medical-coronary care unit of Songklanagarind hospital from 2005 to 2011. Multivariate logistic regression analysis was used to identify predictors of mortality.

## Results

A total of 1,048 patients admitted to ICU for septic shock was divided into two groups: in-ICU survivors (*n *= 555 (53%)) and nonsurvivors (*n *= 493 (47%)). Median survival time was 10 days (95% CI: 8 to 12 days). The respiratory tract was the most common site of infection (47.6%). Community-acquired infections accounted for 59.6%. Survivors were older than nonsurvivors (62 vs. 56 years, *P *= 0.016). Nonsurvivors were more severely ill and had shorter ICU stays (2 vs. 5 days, *P *< 0.001). Nonsurvivors received albumin and steroid more than survivors. Median cumulative fluid at 24, 48 and 72 hours of septic shock onset were 4.2, 7.7 and 10.5 l respectively. Nonsurvivors had significantly larger median cumulative fluid intake at 24 hours (4.6 vs. 3.9 l, *P *< 0.001), at 48 hours (8.2 vs. 7.1 l, *P *< 0.001) and at 72 hours (11.4 vs. 9.9 l, *P *< 0.001). Nonsurvivors also had significantly larger fluid balance (5.4 vs. 4.4 l, *P *< 0.001) and mean fluid balance (2.8 vs. 1.6 l, *P *< 0.001) within 72 hours. In multivariate logistic regression analysis, factors significantly associated with ICU mortality were mean fluid balance, APACHE II score, SOFA score, length of ICU stay, ARDS, steroid use, parenteral nutrition use and source of infection.

## Conclusion

A more positive cumulatively fluid balance over 3 days is associated with ICU mortality in septic shock. Multivariate analysis found not only nonmodifiable factors such as severity score, source of infection, length of ICU stay and ARDS, but also modifiable factors such as parenteral nutrition use, steroid use and mean fluid balance were significantly associated with mortality.

